# Evaluating online health information utilisation and its psychosocial implications among breast cancer survivors: Qualitative explorations

**DOI:** 10.34172/hpp.42682

**Published:** 2024-03-14

**Authors:** Samar J Melhem, Shereen Nabhani-Gebara, Reem Kayyali

**Affiliations:** ^1^Department of Biopharmaceutics and Clinical Pharmacy, School of Pharmacy, The University of Jordan. Amman-Jordan; ^2^Department of Pharmacy, School of Life Sciences, Pharmacy and Chemistry, Kingston University London, Kingston upon Thames, Surrey KT1 1LQ, UK

**Keywords:** Anxiety, Breast neoplasms, Cancer survivors, Health communication, Information seeking behaviour, Internet, Patient education as topic, Psychosocial factors

## Abstract

**Background:**

This study investigated the online information-seeking behaviours of breast cancer patients at Jordan University Hospital, focusing on their dissatisfaction with available online health resources and its impact on their well-being and anxiety levels.

**Methods:**

Employing descriptive phenomenology and convenience sampling, we conducted five Skype-based focus groups with 4-6 breast cancer survivors each, from March to July 2020. Data analysis was performed using NVivo, following Braun and Clark’s inductive thematic analysis framework.

**Results:**

The thematic analysis revealed critical insights into survivors’ interactions with online cancer resources, identifying key subthemes such as the quality of online information, cyberchondriasis, health literacy and search strategies, the distress caused by counterproductive searches, and the tendency to avoid internet searches.

**Conclusion:**

The study underscores the challenges breast cancer survivors face in accessing online health information, especially in Arabic. It highlights the need to improve the quality and accessibility of these resources. Enhancing the cultural relevance of online materials and educating patients on effective information evaluation are crucial. These measures can significantly boost health literacy, mitigate anxiety, and provide better support for breast cancer survivors.

## Introduction

 Breast cancer, a complex disease, often leaves patients in a state of confusion and with an intensified need for information.^1-3^ Even before starting their treatment journey, these patients commonly become proactive information seekers.^4-6^ Despite appreciating the internet as a significant source of information, just trailing behind healthcare professionals, they predominantly rely on their healthcare providers for crucial cancer-related knowledge.^7,8^ Pre-treatment consultations typically encompass 12 to 20 questions per patient,^9,10^ underscoring the paramount importance of precise and comprehensive information in aiding decision-making and ensuring patient-centred care.^11,12^

 However, with the growing number of cancer survivors, the current healthcare system faces challenges in addressing their evolving needs.^13^ Consequently, the responsibility of procuring health information and managing symptoms increasingly falls onto the survivors themselves.^14^ This situation exists despite the abundance of health information, as many survivors report experiencing a gap in crucial information.^15^ This deficit leads them to frequently turn to online resources as their secondary preferred source of health-related information.^15,16^ Nevertheless, an alarming trend persists: a significant number of breast cancer patients express dissatisfaction with the information they receive.^17^ This dissatisfaction detrimentally impacts their quality of life,^18^ functional capacity, and emotional well-being,^17,18^ and may amplify their levels of anxiety.^19^ It can also foster regrets about the treatment decisions made.^20^ Notably, the manner in which individuals seek information online may alter over time, potentially leading to a state of information stagnation or inertia. Conversely, when patients’ information needs are met effectively, they experience enhancements in their quality of life,^18,21^ mental health,^22^ and report diminished levels of anxiety and depression.^23,24^

 Cultural nuances also shape the information needs of breast cancer patients.^15,25-27^ A considerable gap exists in understanding how Arabic-speaking breast cancer patients, part of the approximately 440 million Arabic-speaking population who utilise the Arabic internet, interact with digital resources throughout their cancer care.^28^ Scrutinising these experiences could potentially augment the quality of breast cancer care, and contribute to the refinement of clinical practice guidelines. Only a handful of studies have delved into the online information-seeking behaviors of cancer survivors, exploring their attitudes and experiences towards online cancer-related information, the obstacles they encounter in their quest for pertinent information, and the content they prefer in online health resources.^29,30^

 The main objective of this research is to fill the existing gap in knowledge by examining how Arabic breast cancer survivors seek information online. This study aims to achieve two goals; firstly, to understand the patterns and experiences that breast cancer survivors encounter while searching for cancer-related information online through in-depth focus groups as an approach and secondly to gain insights into the obstacles and challenges faced by breast cancer survivors during their online exploration for cancer-related information.

## Materials and Methods

###  Study design and setting

 Descriptive phenomenology and qualitative methods were chosen for studying the online information-seeking behaviors of cancer patients due to their focus on capturing authentic, real-life experiences.^31^Descriptive phenomenology excels in revealing the core of individuals’ experiences, enabling researchers to deeply understand the subjective views of cancer patients navigating online health resources.^31^ This approach is particularly effective in grasping the subtle complexities and emotional nuances of personal experiences, which are often overlooked by quantitative methods.^31,32^ By employing qualitative methods, the study achieves a richer, more detailed understanding of the patients’ experiences, including their fears, needs, and preferences. Such in-depth insights are vital for the development of more effective, patient-centric online health platforms and tools, tailored to the specific needs and situations of cancer patients. This study was conducted between March and July 2020 to investigate the experiences of individuals who have survived breast cancer while searching for cancer-related information online. We gathered five focus groups, each consisting of 4-6 participants from the outpatient clinics at Jordan University Hospital (JUH). In this paper, we include quotes from breast cancer survivors identified by their focus group number, their position in the group (1-6), and their age.

###  Participant recruitment and enrolment

 This study employed convenient sampling to recruit female breast cancer survivors aged 18 or older. The eligible participants were in stages (0-IV) of breast cancer with or without recurrence, had completed or were undergoing curative treatment, and possessed digital skills and owned a mobile device. We excluded individuals who were under 18 years old, had other types of malignancies, patients who are actively receiving treatment, and patients who lack Arabic or digital literacy skills. A total of 25 women who were not known to the researchers were successfully enrolled and divided into five focus groups.

###  Data collection

 For data collection purposes, a team consisting of three female academics developed a structured interview guide based on an extensive review of relevant literature.^14-17,18,23,30,33-36^

 The reason for selecting the focus group method was its ability to gather information, facilitate comparison and contrast of ideas, and encourage participants to generate ideas. These benefits aligned with the goals of our study.^37^ To ensure we obtained responses that deepened our understanding of survivors’ perspectives and experiences with accessing cancer information, we designed a guide consisting of probing and open-ended questions.

 Due to COVID-19 restrictions, the first author (SJM) conducted the focus groups remotely using Skype. Each session lasted between 120 and 180 minutes. All interactions were recorded in a format. We took steps to anonymize the transcriptions while preserving confidentiality. You can find the set of questions and prompts in Supplementary file 1.

###  Data analysis

**Figure 1:**
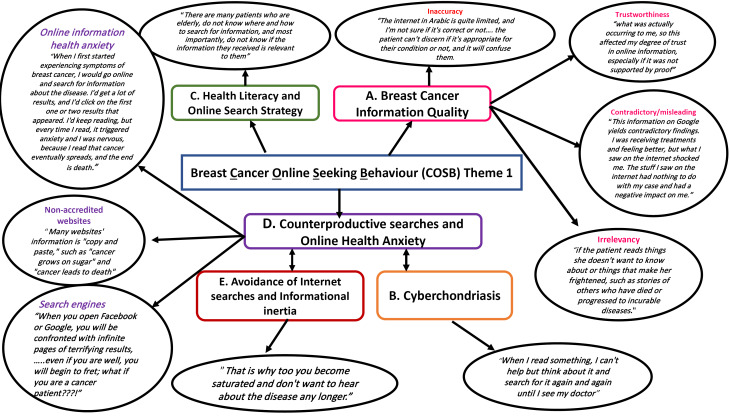
Conceptual map of digital experiences and perceptions of cancer online information behaviour (COSB) based on female breast cancer survivors

 We used NVivo 12 Software is available at Lumivero’s website: https://www.lumivero.com to manage and analyse anonymized transcriptions. Our approach involved Braun and Clarke’s thematic analysis method, which included becoming familiar with the data, coding it, and condensing it into themes.^38^ To ensure validity, we cross-checked all codes against the dataset. Figure 1 presents a map illustrating Cancer Online Information Seeking Behavior (OISB).

###  Quality assurance and rigor

 Quality assurance in this study was upheld through the following the principles of thematic analysis methodology and the iterative development of themes, searching and reviewing the themes and evaluating the themes across the entire data set.^38^ The study’s findings were reported following the Consolidated Standards for Reporting Qualitative Research (COREQ) criteria,^39^ detailed in Supplementary file 2. A flexible interview schedule was utilized to enhance the research’s robustness. To ensure the study’s integrity, transferability, and credibility, methods such as accurate data recording and transcription, including direct quotations for referential adequacy, and maintaining clear, transparent data collection and analysis processes were implemented. Contextual clarity was provided by describing the sample and research settings. Regular collaborative discussions among researchers contributed to the study’s rigor, and findings are presented using a specific format that includes participant and focus group details. The study design, in line with COREQ guidelines, was refined through a pilot interview with two breast cancer survivors, who were not included in the main study. Initial analysis and code generation by the first author were cross-verified by co-authors to ensure thoroughness and accuracy.

## Results

###  Participants characteristics

Table 1 provides information about the 25 participants who took part in the focus group. All participants were women in the follow up stage of care except for one participant who had completed therapies and was waiting for a preventive mastectomy. The median age was 55 years with an age of 46 years. The median survivorship duration, measured from cancer diagnosis to interview time was six years ranging from a minimum of 2 years, to a maximum of 23 years.

**Table 1:** Breast cancer survivors’ characteristics who participated in 5 focus groups

**Variable(s)**	**CRC survivors (N=25)** **No. (%)**
Age (y)^a^	55 (37-73)
Education	
Primary (5-8 years)	2 (8)
Secondary (9-12 years)	6 (24)
High school/collage/diploma (12 + years)	0 (0)
University (14 + )	17 (68)
Employment	
Employed	13 (52)
Unemployed (capable/uncapable)	7 (28)
Retired	5 (20)
Cancer stage	
Stage I	8 (32)
Stage II	14 (56)
Stage III	3 (12)
Stage IV	0 (0)
Survivorship in years	
2-5	13 (52)
5-8	7 (28)
8-11	3 (12)
> 11	2 (8)

^a^ Median (min, max).

###  Thematic analysis: Cancer OISB of breast cancer survivors

 One overarching theme emerged from the entire thematic analysis of focus groups on breast cancer on online seeking behavior of breast cancer survivors (Figure 1) which focuses on breast cancer survivors’ perceptions and interactions with online cancer resources, the theme is broken down into five subthemes, discussed below, the subthemes constitute the factors influencing online seeking behavior and potential barriers, attitudes, and ramifications pertaining to breast cancer information quality on the Internet. Figure 1 shows a conceptual map of digital experiences and perceptions of OISB of breast cancer survivors.

###  A. Breast cancer online information quality (Inaccurate, misleading, incomplete, irrelevant, non-trustable)

 Breast cancer survivors frequently struggle with the lack of clarity surrounding the validity, accuracy, and applicability of health information. This uncertainty can stem from inconsistent or complex information, misinformation, or a dearth of evidence-based content, leading to confusion or misinterpretations. Such ambiguity, essentially characterising cancer-related information, can cast doubts on its integrity, comprehensiveness, relevance, and trustworthiness, causing significant distress. This uncertainty can compromise trust in online cancer resources, escalating feelings of anxiety and fear. For instance, a patient seeking information about “chemo fog” or “chemo brain” might find themselves overwhelmed with excessive data, heightening their tension and apprehension. Therefore, it is paramount that health information, made available to survivors, is personalised by the physician, so it only aligns with their unique circumstances thus offering them a sense of reassurance.

 “*For example, I go to a website to learn more about Chemo fog or Chemo brain because it happened to me after the sixth chemo round……. the online information I found by Google provides far more detail than is required. You may become stressed and freaked out as a result of what you read. That’s why it’d be much better if you had the information from the doctor; it’ll be more relevant and provide you peace of mind, because doctors give you what you need. While the Internet will show you the most dreadful consequences or may tell you that this is irrevocable, leaving this to the patients may freak you out” *(Focus group 1, breast cancer survivor 2, 37 years).

 “*The drawbacks may include, for example, if the patient reads things she doesn’t want to know about or things that make her frightened, such as stories of others who have died or progressed to incurable diseases” *(Focus group 2, breast cancer survivor 4, 55 years).

 “*Yes, there was a mismatch between what I looked for and what was actually occurring to me, so this affected my degree of trust in online information, especially if it was not supported by proof, and there is no single source on which we can rely” *(Focus group 5, breast cancer survivor1, 38 years).

 “*The Internet in Arabic is quite limited, and I’m not sure if it’s correct or not; because when you google anything, you are swamped with material, and the patient can’t discern if it’s appropriate for their condition or not, and it will confuse them” *(Focus group 3, breast cancer survivor 6, 57 years).

 “*This information on Google yields contradictory findings. I was receiving treatments and feeling better, but what I saw on the Internet shocked me. The stuff I saw on the Internet had nothing to do with my case and had a negative impact on me” *(Focus group 4, breast cancer survivor 4, 69 years).

 “*On the other hand, if the individual does not know the details of their disease, they may get anxious and afraid if the information is not personalized, therefore the patient must be knowledgeable of their diagnosis” *(Focus group 1, breast cancer survivor 1, 44 years).

 “*Of course, this varies from person to person because not everyone has the same level of information or requirement, but most people will begin gathering information about the condition at the early onset of diagnosis, which can cause a lot of fear and confusion because we don’t know what is right and wrong and don’t know our exact diagnosis” *(Focus group 1, breast cancer survivor 1, 44 years).

 Furthermore, scientific cancer related Internet content in Arabic is fairly limited, thus providing a language barrier to effective searches as most information is in the English language. The language barrier may also make it difficult to determine whether the information is relevant to the specific disease condition and patients’ needs. In addition, there are contradicting findings available online, which might have a negative impact on the patient. For example, if the individual does not know the specifics of their illness, they may get nervous and fearful. This is especially true if the patient is unaware of their diagnosis. As a result, some patients may decide to discontinue “Googling” breast cancer information because it makes them feel worse. In addition, if the patient listens to several sources, they may not know which one to trust, which can hinder treatment.

 “*Also, if I listen to multiple sources, it will interfere with my treatment. For example, should I listen to my doctor or consider what I learn on the Internet, given that cancer is cancer!!!” *(Focus group 5, breast cancer survivor 2, 73 years).

###  B. Cyberchondriasis

 A segment of participants responses may reflect cyberchondriac behavior. A few survivors who exhibited higher levels of anxiety, spent longer and performed repeated online searches, they had obsessive thoughts and misinterpretations about their health, and sought cancer-related material more frequently. It is important to note that this behavior can be detrimental to one’s mental health and can lead to further distress. Therefore, it is important for patients to avoid using online resources for diagnosis or to learn about the disease. Instead, better approach is to talk to the doctor who is treating them as the best way to gain information.

 “*When I read something, I can’t help but think about it and search for it again and again until I see my doctor, so the patient should avoid looking online for self-diagnosis or even learning about the disease in the early stages of diagnosis because it will add more distress and may do no good at all. Talking to the doctor who is treating you is the best way to get accurate information and to calm yourself down” *(Focus group 3, breast cancer survivor 3, 54 years).

 “*I find myself in a loop of verifying information, leading to heightened anxiety. I endeavour to stop my searches, but my worries persist. I return to the Internet seeking more knowledge, especially when a particular diagnostic test is suggested, or when healthcare professionals express a concern without specifying it...” *(Focus group 4, breast cancer survivor 3, 42 years).

 “*In the beginning, when symptoms started showing before the diagnosis, I would consult other patients. It was a truly harrowing period, and constant information-seeking became an insomnia-inducing obsession, only compounding my distress... This pattern persisted until I was diagnosed, plunging me into a profound state of denial. The surgeon’s insistence on surgery was particularly hard-hitting, as I wrestled with fears of how it could shatter my identity as a woman... I was in constant pursuit of information that could bolster my denial of having cancer... That was just the first stage... but, honestly, it was a cycle that kept repeating... Especially post-surgery, when they start discussing chemotherapy and what comes next... it was a never-ending source of distress.” *(Focus group 1, breast cancer survivor 2, 37 years).

###  C. Health literacy and online search strategy

 One of the Internet’s distinguishing characteristics is that anybody can potentially disseminate health-related material, resulting in a lack of expert oversight and limited accuracy of information. Therefore, individuals seeking health information regarding cancer online face several challenges, as they are required to actively participate in the evaluation of a massive volume of frequently unverified online health data. Thus, patients who struggle with analysing online medical data may be exposed to inaccurate or insufficient information, which may have been linked to negative health outcomes such as poor decision-making or treatment adherence. Survivors’ ability to analyse available cancer information on the Internet is certainly a problem that demands more attention and that has not been addressed thus far. Further, low health literacy and an imprecise search approach are also important risk factors for search results that could result in misunderstandings.

 “*Many times, a person searches for information on Google and receives numerous results, many of which are from websites that may be unsupervised by specialists, so all you get sometimes is incorrect information... because there are many people who do not have a health background or who are not specialists such as a nurse or pharmacist, so they are misled by these websites that provide inaccurate information.” (Focus group 4, breast cancer survivor 3, 42 years) *

 Chemotherapy patients, who often have limited education and experience, often turn to the Internet for more information. Participants claimed that even professionals with a health background, such as nurses or pharmacists, might be misled by false material on these websites. Besides, many older patients don’t know where or how to seek for information, and most critically, they don’t know if it’s relevant to them.

 “*There are many patients who are elderly, do not know where and how to search for information, and most importantly, do not know if the information they received is relevant to them*” (Focus group 5, breast cancer survivor 5, 43 years).

 A participant of the study, possessing a medical background, emphasised the significance of DHL. Her proficient understanding of medical terminology, attributed to her professional specialty, gave her a potential advantage to execute health-related Internet searches.

 “*The fact that I am a nurse may help me better comprehend medical material….” *(Focus group 4, breast cancer survivor 1, 59 years).

 To address this issue, the informants proposed that an app or website that gathers all the relevant information in one location will help cancer patients avoid mistakes when searching for cancer-related topics. This would lessen misunderstanding and confusion when searching for medical information online. Such an app or website would also benefit patients who are not competent at searching by providing all the relevant information in one location without overwhelming them.

 “*If there were an app or website that combined all of this information, cancer patients would be less likely to make mistakes while searching for a cancer related topic. This is especially true during chemotherapy, when patients lack knowledge and rely on search engines like Google for information. I had no idea whether this was a scientific source, and as a result, patients, like me become confused” *(Focus group 3, breast cancer survivor 3, 54 years).

###  D. Counterproductive online searches cause anxiety, distress and fear

 Participants responded that engaging in counterproductive internet searches aggravated their discomfort. This is due to non-accredited websites. Additionally, a cancer diagnosis imposes a genuine and persistent threat that patients frequently struggle to adjust to, all the more so because there is no “reassuring sign” indicating that the threat has passed. Even after the disease has stabilised, concerns of death are widespread, and it has been argued that they contribute to anxieties of progression. As a result, emotional fragility and health-related anxiety remain dormant factors that can be readily activated while conducting ineffective searches and visiting several websites that expose users to untailored reassuring informational material.

 “*When it comes to cancer, patients are emotionally fragile, so, contrary to Google search and online cancer information on the Internet, if a platform is available, its content should be formulated in a way that does not provoke negative emotions or trigger fear from the illness. It should be done in an honest and scientific manner, and for sensitive issues, we should rely on direct communications with the treating doctor. And it should be as stage specific as possible; for example, if I am in stage 2, I don’t want to read about stage 3 cancer” *(Focus group 1, breast cancer survivor 2, 37 years).

 Another informant said: *“When you open Facebook or Google, you will be confronted with infinite pages of terrifying results. Even if you are well, you will begin to fret; what if you are a cancer patient???!” *(Focus group 5, breast cancer survivor 2, 73 years).

 Others indicated:* “As you are aware, cancer is not a simple condition, so you must trust your doctor and not rely on information from other sources. Going on a search here and there will just add to your anxiety, confuse you, and divert you” *(Focus group 2, breast cancer survivor 3, 54 years).

 “*Many websites’ information is “copy and paste,” such as “cancer grows on sugar” and “cancer leads to death,” so when you see this statement repeated time and time again, all you need to do is shut down your head and only listen to the doctor” *(Focus group 3, breast cancer survivor 6,57 years).

 This was echoed by others who indicated:* “When I first started experiencing symptoms of breast cancer, I would go online and search for information about the disease. I’d get a lot of results, and I’d click on the first one or two results that appeared. I’d keep reading, but every time I read, it triggered anxiety and I was nervous, because I read that cancer eventually spreads, and the end is death” *(Focus group 4, breast cancer survivor 3, 42 years).

 “*I learn from them, but I am not very good at searching; sometimes I get a lot of results when I click on something; perhaps this is the problem; I get overwhelmed by too much information” *(Focus group 5, breast cancer survivor 2, 73 years)

 “*So, I don’t know where to seek for information, so I just google what I need to look for, which only makes me more scared and causes me to worry about it all the time” *(Focus group 4, breast cancer survivor 4, 69 years). 

###  E. Avoidance of internet searches and information inertia

 It is comprehensible that the majority of participants said that the inefficiency, ambiguity, and volume of online information, combined with the physical distress, and anxiety-induced, and the potential interference of the content with their treatment decision, contributed to their avoidance of Internet searches thus leading to informational inertia. Information on the Internet can be overwhelming and confusing, hence causing stress.

 “*I had a dreadful experience with web searches since I read a lot about that illness and was already overwhelmed, so I didn’t need to continue reading when the material was deemed untrustworthy, and everything I got was dismissed by doctors” *(Focus group 1, breast cancer survivor 1, 44 years).

 Another informant said:* “So, I stopped Googling breast cancer information since it makes me feel worse. I simply do not believe them” *(Focus group 5, breast cancer survivor 2, 73 years).

 Because there is so much information available, it can be challenging to evaluate which sources are genuine and trustworthy, hence leading individuals to stop searching for information about the disease or treatment side effects.

 “*Honestly, when I looked for disease information by searching, the results can be confusing and I got muddled up, anytime I read any information I obtained, I felt frightened and disturbed, and I started thinking that cancer would come back!!! That’s why I’ve stopped looking for cancer-related information on the Internet; it simply makes me feel worse” *(Focus group 2, breast cancer survivor 1, 52 years).

 “*That is why too much information is harmful; you become saturated and don’t want to hear about the disease any longer” *(Focus group 2, breast cancer survivor 1, 52 years).

 “*I agree with others who prefer not to read about the side effects because I may misinterpret the signs and symptoms, causing health anxiety and concern. This can happen to a lot of patients, especially if you seek for information on the Internet” *(Focus group 4, breast cancer survivor 1, 59 years).

## Discussion

 This study identified multiple challenges encountered by Arabic-speaking breast cancer survivors in their pursuit of online information. These challenges include the dubious quality of online resources and language barriers, the risk of ‘cyberchondriasis’, and the need for improved health literacy and search strategies. Often, counterproductive searches lead to anxiety and fear, causing many participants to avoid online information due to its overwhelming and ambiguous nature, resulting in information inertia.

 The study observed a common pattern among breast cancer survivors: their online information search is frequently marred by significant health anxiety. This aligns with a 2020 meta-analysis by Hashemi et al,^40^ which noted a high incidence of anxiety among this demographic. Their analysis of 36 studies from 2000 to 2018, encompassing 16 298 patients, revealed that 41.9% (95% CI: 30.7, 53.2) experienced anxiety, with a higher prevalence in Mediterranean countries. Thus, online health information anxiety is a facet of the broader spectrum of anxiety that may be prevalent in this group.

 Our findings highlight that ineffective search strategies and poor information quality contribute to fluctuations in online information-seeking across the care trajectory. This observation was echoed by Perrault et al,^41^ who found that breast cancer patients often use a wide array of search terms. Consequently, cancer educators should ensure that websites include these terms in titles, URLs, and overview texts that appear on search results pages.^41,42^ A topic-organized search strategy guide linked to relevant websites could help breast cancer survivors and newly diagnosed patients navigate this complex information landscape. Our findings suggest the need for these modifications across various online language landscapes to guide patients towards more factual and understandable treatment information.

 Online resources can aid cancer patients in understanding their condition, especially when uncertainty exists or before seeking medical advice. They can also help in adjusting, building confidence, and making informed decisions post-diagnosis.^41,43^ However, our study found that the online health search experiences of breast cancer survivors were characterized by health concerns and emotional discomfort. The information on Arabic-language websites was often of poor quality, with inaccuracies, irrelevant searches, and misleading results contributing to their anxiety and fear.^44,45^ Internet-based medical information research can exacerbate uncertainty and health anxiety, especially in individuals with low tolerance for uncertainty. Health anxiety is further heightened by the tendency to overreact to ambiguous online health information.^46^ This can lead to cyberchondriasis, defined as excessive or repetitive online health information searches that increase concern or distress about one’s health.^47^ The ambiguity and often misleading nature of Internet health information exacerbate this distress and anxiety, creating a feedback loop.^47,48^

 Anxiety is common in cancer patients, and the model proposed by Curran et al^49^ illustrates how clinically significant anxiety can develop during diagnosis, treatment, and follow-up. It applies to patients who have completed treatment but continue to have the disease, as well as those not in remission who require ongoing treatment or monitoring. Anxiety is influenced by pre-existing beliefs, previous cancer experiences, intolerance for uncertainty, and meta-cognitive perceptions of anxiety. Continuous vigilance or avoidance of cancer-related stimuli, coupled with attempts to regain control, can heighten anxiety, especially when combined with systemic factors that can either mitigate or exacerbate it. Therefore, content developers should consider subtle factors like the patient’s stage in the care continuum, their anxiety level, and their tolerance for uncertainty when creating digital content and psychoeducational interventions for breast cancer survivors. Tailoring the volume and type of information to the individual’s needs is crucial for optimizing the search strategy and can improve care quality and overall wellbeing.

 The majority of evaluation studies conclude that the quality of health information on the Internet is questionable.^50^ In 2018, Alnaim^51^ evaluated Arabic websites offering breast cancer information and found significant insights. A notable portion of these websites were either commercially operated (47%) or run by non-profit organizations (33%). While they covered a range of topics related to breast cancer, none discussed complementary medicine. Approximately two-thirds (67%) were deemed to provide entirely accurate information. However, only five websites were attributed to healthcare professionals, with nine lacking clear authorship. Despite the abundance of websites, there is a significant gap in comprehensive surgery information for Arabic-speaking women. The study highlighted the urgent need to improve the accessibility and quality of online information, which would greatly enhance the patient experience. The majority of Arabic cancer videos on YouTube, like much Internet content, are not peer-reviewed, allowing anyone to publish content. Videos created by non-specialists garner the most views and likes. Additionally, the comments on these videos reveal insufficient e-health literacy and a lack of clear online health strategy, with viewers generally unable to distinguish between evidence-based material and false content.^52,53^

## Conclusion

 This study highlighted the critical link between online information-seeking behaviour, health anxiety, and the quality of online health information for breast cancer survivors. It demonstrates how inadequate search tactics and low information quality might worsen health anxiety, especially among frequent online health information seekers. The findings urge for improved Arabic-language health resources that address the unique needs of Arabic-speaking breast cancer survivors.

## Strengths and Limitations

 In this study, qualitative methodologies were employed, offering detailed insights but encountering limitations in terms of generalizability and potential researcher biases stemming from the interpretive nature of the analysis. Despite these constraints, qualitative research is distinguished by its ability to delve deeply into complex phenomena. It is particularly effective in examining the influence of social and cultural factors on individual experiences. This methodology is crucial for uncovering underlying motivations and sociocultural impacts, which are often overlooked in quantitative approaches. Consequently, the insights gained through this qualitative lens are instrumental in informing the development of nuanced interventions and policies, tailored specifically to meet the unique needs of distinct and comparable populations.

## Practical implications

 This study highlights the need for accessible, high-quality online health resources and addressing health anxiety among breast cancer survivors. It deeply examines the digital experiences of these survivors, providing a framework for creating relevant digital content. This research is invaluable for researchers, clinicians, and educators, guiding the development of effective online resources and strategies to mitigate health-related anxiety.

## Future research directions

 Future research should prioritise the exploration of strategies aimed at enhancing the quality of health information available online, particularly in relation to breast cancer online platforms. Additionally, it is crucial to investigate the involvement of healthcare providers and information specialists in educating patients about effective practices for seeking reliable information. Furthermore, investigating the elements that contribute to online information anxiety or Cyberchondriasis could help to lead the development of interventions for individuals or specific time periods linked with uncertainty or elevated levels of health anxiety. Hence the study emphasises psychoeducational support for breast cancer survivors’ high anxiety rates.

## Acknowledgements

 The authors wish to thank to the University of Jordan and Jordan University Hospital for their support in the recruitment process and research phase.

## Competing Interests

 The authors have no competing interests to disclose.

## Ethical Approval

 The research received approval from the Institutional Review Board of JUH (10/2019/8990) and Kingston University’s Ethical Committee (Protocol no. 1416). We ensured both written and verbal informed consent from all participants, prioritizing their privacy and confidentiality. This manuscript is a qualitative study and excludes any identifiable participant information. Participants were fully briefed on the study’s aims and methods as detailed in the participant information sheet (PIS), and institutional consent was secured through a formal consent form. The PIS is included as an additional document.

## Supplementary Files


Supplementary file 1. Focus Group Topic Guide for Investigating Digital Experiences of Breast Cancer Survivors: Online Information Seeking Behavior (OISB)

Supplementary file 2. Consolidated Criteria for Reporting Qualitative Research (COREQ) 32-item checklist Breast cancer survivors focus groups
