# Supplementary material for: Evaluating online health information utilisation and its psychosocial implications among breast cancer survivors: Qualitative explorations
Source: Health Promot Perspect. 2024 Mar 14;14(1):61–9. doi: 10.34172/hpp.42682 (PMC11016143; doi:10.34172/hpp.42682)
Supplement: Supplementary file 1 — Focus Group Topic Guide for Investigating Digital Experiences of Breast Cancer Survivors: Online Information Seeking Behavior (OISB) [file hpp-14-61-s001.pdf]

Melhem et al, **Health Promotion Perspectives**, 2024, 14(1), 61-S1
doi: 10.34172/hpp.42682
https://hpp.tbzmed.ac.ir

Supplementary 1: Focus Group Topic Guide for Investigating Digital Experiences of Breast Cancer Survivors: Online Information Seeking Behavior (OISB)

Opening Question(s)

1.**Digital Exploration and Motivation**:
- Can you share your initial experiences in seeking cancer-related information online after your diagnosis? What motivated you to look for information in this way?
  - Probe: Tell me why you chose the digital medium over others.
  - Probe: How did your emotional state influence your search strategies?

Search Strategies and Information Quality

2. **Search Strategies:**
- Describe how you went about searching for cancer-related information online. What specific strategies did you use to find the information you needed?
  - Probe: How did you decide which sources were trustworthy?
  - Probe: Can you give examples of keywords or phrases you frequently used?

3. **Quality of Information**:
- In your search for cancer-related information, how did you assess the quality of the information you found? Did these sources meet your needs?
  - Probe: Explain more about any criteria you used to judge information quality.
  - Probe: What about the information you found was particularly helpful or unhelpful?

Information Inertia and Peak Seeking

4. **Information Seeking Behavior Over Time:**
- Was there a period during your journey when you found yourself looking for information more intensively? When was this, and why do you think that was the peak?
  - Probe: Did you experience information overload or fatigue? How did you overcome this barrier?

5. **Discontinuation of Information Search:**
- Was there a point at which you stopped looking for information online? What prompted this decision?

- Probe: Tell me why you might have felt overwhelmed or satisfied with the information you had.

Barriers and Support Needs

6. **Barriers in Information Seeking**:
   - What barriers did you encounter while searching for cancer-related information online?
      - Probe: How did you overcome these barriers?
      - Probe: What about other participants' ideas or strategies that you heard about and found useful?

7. **Emotional and Psychological Support**:
   - How well do you feel the available information sources cater to the emotional and psychological support needs of breast cancer survivors?
      - Probe: Can you share experiences where the information impacted your emotional well-being positively or negatively?

Language-Specific Needs and Gaps

8. **Availability and Quality of Arabic Language Resources:**
   - Discuss the availability and your evaluation of cancer-related information in Arabic. Do these sources fulfill your information needs, especially regarding psychological and emotional support?
      - Probe: What gaps have you noticed in the availability of quality information in Arabic?
      - Probe: How could these resources be improved to better support Arabic-speaking survivors?

Closing Questions

9. **Overall Experience and Suggestions:**
   - Reflecting on your entire journey of seeking information online, how would you describe your overall experience? What improvements or changes would you suggest to make these digital resources more effective for breast cancer survivors?
      - Probe: What advice would you give to someone just starting their search for cancer-related information online?
