# Supplementary material for: Evaluating online health information utilisation and its psychosocial implications among breast cancer survivors: Qualitative explorations
Source: Health Promot Perspect. 2024 Mar 14;14(1):61–9. doi: 10.34172/hpp.42682 (PMC11016143; doi:10.34172/hpp.42682)
Supplement: Supplementary file 2 — Consolidated Criteria for Reporting Qualitative Research (COREQ) 32-item checklist Breast cancer survivors focus groups [file hpp-14-61-s002.pdf]

Melhem et al, **Health Promotion Perspectives,** 2024, 14(1), 61-S2
doi: 10.34172/hpp.42682
https://hpp.tbzmed.ac.ir

Supplementary 2: Consolidated Criteria for Reporting Qualitative Research (COREQ) 32-item checklist Breast cancer survivors focus groups

Developed from:

Tong A, Sainsbury Craig J. Consolidated criteria for reporting qualitative research (COREQ): a 32-item checklist for interviews and focus groups. *International Journal for Quality in Health Care.*2007, Volume 19, Number 6: pp.349-357

COREQ Criteria	Criteria fulfilment in the current research
**Domain 1: Research team and reflexivity**	
**Personal Characteristics**	
1. Interviewer/ Facilitator	It was noted in the methodology section that the first author (SJM) performed the focus groups
2. Credentials	In the research paper, the research team's qualifications and affiliations were listed.
3. Occupation	The research team's occupations were given in the affiliations section.
4. Gender	The methods section noted that the analysis team comprised of three female academics.
5. Experience and training	The analysis was undertaken by a research team with rich experience performing qualitative research in healthcare settings, as stated in the methods section.
**Relationship with participants**	
6. Relationship established	This was not stated in the study. Before the study, none of the research team had relationships with any subjects.
7. Participant knowledge of the interviewer	SJM phoned all eligible participants to clarify the research objectives and answer any questions, and because the interviewer and breast cancer survivors did not know each other, additionally a brief explanation of the research was given during skype based online focus groups. The participants were informed of the study's objectives prior to the Skype focus groups. Researchers emailed or WhatsApped participants a research information sheet. This was in methodology section.
8. Interviewer characteristics	Qualification, occupation, gender, and the absence of a pre-existing link between the interviewer and the interviewees are all provided about the interviewer. The methods section addressed this point.

**Domain 2: Study Design**	
**Theoretical Framework**	
9. Methodological orientation and theory	Descriptive phenomenology was the main theoretical orientation. Data was analysed, however, using inductive thematic analysis. This was noted in the methods section.
**Participant selection**	
10. Sampling	A convenience sampling strategy of ambulatory breast cancer survivors was used, with participants recruited from a large tertiary hospital (JUH) utilising an indirect recruitment strategy by their specialists, as detailed in the methods section.
11. Method of approach	Clinicians approached participants in person or by phone. This was stated in methodology.
12. Sample size	This study included 25 breast cancer survivors who were interviewed. As stated in the methodology section, sample size was chosen by data saturation and a stopping threshold of three interviews. five focus groups (comprising 25 survivors) were held, and talks were halted when no new fresh ideas for topics were created, indicating thematic saturation.
13. non-participation	All eligible participants who were approached and agreed to participate in accordance with the procedures described in the methods section were enrolled. A flow chart outlining the recruitment process for breast cancer survivors was included in the methods. Thus, the chapter did not discuss non-participation.
**Setting**	
14. Setting of data collection	Focus groups with breast cancer survivors were held through Skype. This was mentioned in methodology.
15. Presence of non-participants	Nobody other than the participants in the focus groups was present.
16. Description of sample	Table 1 shows the sample characteristics of breast cancer survivors, Mentioned in the results of research paper .
**Data Collection**	
17. Interview guide	The topic guide was provided in supplementary 1.
18. Repeat interviews	There were no follow-up interviews, as noted in the methods section.
19. Audio/ visual recording	As stated in the methods, all online focus groups conducted via Skype were audio-recorded.
20. Field notes	No handwritten notes were taken.
21. Duration	The length of the online focus groups was specified in the methodology section.
22. Data saturation	Saturation of data was used to determine sample size, and this was documented in the methods section.

23. Transcripts returned	For participants to review and remark on, transcripts were not given back.
**Domain 3: analysis and findings**	
**Data analysis**	
24. Numbers of data coders	The lead investigator SJM performed data coding. However, the two co-authors separately inspected the original first author's coding, and disagreements were discussed. All authors thoroughly debated and approved the coding framework.
25. Description of the coding tree	The "Methods" section describes the code structure and conceptual map.
26. Derivation of themes	Themes were derived inductively from data and literature review.
27. Software	The NVivo 12 Software is available at Lumivero's website: https://www.lumivero.com was used to manage and code the data.
28. Participant checking	This was not carried out.
**Reporting**	
29. Quotations presented	Under the results section, participants' quotes were included.
30. Data and findings consistent	To verify interpretations and findings, the author and the supervisors evaluated and validated the final themes and subthemes.
31. Clarity of major themes	The thematic analysis weighted all themes equally.
32. Clarity of minor themes	Within the thematic analysis, all themes received equal weighting.
